# Statistics of Small-Pox

**Published:** 1874-04-15

**Authors:** H. Gradle


### STATISTICS OF SMALL-POX.

REMARKS ON A REPORT OF DR. L. J. KELLER, IN THE "ALLG. WIENER MED. ZEITUNG," BY H. GRADLE, M.D.

AS physician-in-chief of all Austrian government railroads, Dr. L. J. Keller has certainly enjoyed excellent opportunities of observing the result of disease ravaging in the large community entrusted to his care; and this is the second year that a report on this particular affection, variola, has been issued. But as the number of cases observed during 1872 was but limited (573), compared with the experience of 1873, and the relative figures obtained identical, we shall limit our attention to the epidemic of last year:

The Austrian railroads are dependent on about 37,000 employes, which, with their families, constitute an army of 55,000 to 60,000 persons, under the supervision of 80 physicians. Of this number, during the year 1873, the loathesome disease attacked 2,054 individuals, of whom 385 died—a mortality of 18.74 per cent. Classifying these cases, we find amongst them—

| | | | | | |
|---|---|---|---|---|---|
| 1337 | persons never vaccinated, | with | 219 | deaths, or a mortality of | 16.38 ℘c. |
| 596 | persons once vaccinated, | with | 148 | deaths, | 24.83 |
| 46 | " re-vaccinated, | " | 7 | " | 15.22 |
| 11 | " previously attacked, | " | 2 | " | 18.18 |
| 64 | " unknown, | " | 9 | " | 14.06 |

The prominent feature of this table is the large number of cases previously vaccinated; but, unfortunately, we are not told what per centage of the entire population have undergone this operation, though certainly the greater. At any rate, immunity against a subsequent attack seems less perfect than ordinarily supposed [unless vaccination had been carelessly performed?—*Translator*]. But how about the greater fatality—24.83 per cent., versus 16.38 per cent.—of the non-vaccinated individuals? A critical analysis reveals this apparent fact as unreal, at the same time pointing to the necessity of accurate record of all circumstances connected with the case, especially age, to render statistics valid. Not desiring to republish all tables of Dr. Keller, we call attention but to the following:

| | VACCINATED. | | | NON-VACCINATED. | | |
|---|---|---|---|---|---|---|
| Age. | Attacked. | Died. | Mortality. | Attacked. | Died. | Mortality. |
| 1 yr. | 43 | 26 | 60.46 | 168 | 76 | 45.23 |
| 1–2 | 37 | 20 | 54.05 | 63 | 24 | 38.09 |
| 2–3 | 31 | 11 | 35.48 | 35 | 6 | 17.17 |
| 3–4 | 51 | 12 | 23.53 | 55 | 11 | 20.00 |
| 4–5 | 48 | 12 | 25.00 | 54 | 8 | 14.81 |
| 5–10 | 207 | 42 | 20.29 | 85 | 6 | 7.06 |
| 10–15 | 164 | 10 | 6.09 | 35 | 5 | 14.28 |
| 15–20 | 238 | 15 | 6.05 | 32 | 2 | 6.25 |
| 20–30 | 277 | 23 | 8.66 | 39 | 4 | 10.25 |
| 30–40 | 157 | 24 | 15.25 | 21 | 3 | 14.28 |
| 40–50 | 55 | 11 | 20.00 | 4 | 1 | 25.00 |
| 50–60 | 23 | 9 | 39.01 | 3 | 1 | 33.33 |
| 60–70 | 6 | 4 | 66.66 | 2 | 1 | 50.00 |
| | 1337 | 219 | 16.38 | 596 | 148 | 24.83 |

The immense influence of age is manifest in the figures. According to them, 10 to 20 years is the most favorable period, as regards the prognosis, while towards either extreme a rapidly increasing ratio of death is the rule; and especially fatal is the affection in the first two years of life. The mortality at different periods, of the class not vaccinated, is, if anything, smaller than in the other category; but whence the difference in the summary? On taking the average fatality of all cases above two years of age, 13.76 per cent. presents the mean number for vaccinated persons, and 13.15 per cent. for those not enjoying that advantage (?); so that above this period Dr. Keller believes he has proven the inability of vaccination to avert an unfavorable result; but, looking at the table, the difference in the number of deaths below this age is *also* found in favor of the non-vaccinated, *while the much greater number —231 unprotected, to 80 vaccinated children—of a class of individuals with feeble powers of resistance, will, of course, raise the mortality of a given number of cases in which they are included.* This is much more evident on stating the proportion of such young subjects in either class, viz.: 80 in 1337, or 5.98 per cent. of the vaccinated individuals—231 in 596, or 37.08 per cent. of the other—were below two years of age, and *this for the reason that comparatively few children* are vaccinated *below that period.*

Accepting these statements as faithful and accurate, Dr. Keller's conclusions seem justifiable, viz.: that vaccination gives neither immunity, *en masse*, against the disease for which it is employed, nor does it render smallpox less dangerous—the apparent mildness of the affection, in the "protected" persons, being caused by inevitable circumstances, dependent on the law of nature, that the constitution, at the tender age of one and two years, possesses less resistance to disease than at a more advanced period. Finally, he considers vaccination useless. Re-vaccination, likewise, showing a mortality of 15.22 per cent., shares no better fate in the author's opinion, since the absence of any children under four years of age amongst the number precludes the admission of a normally high rate of fatality.

In conclusion, the immunity afforded by a previous attack of variola, seems a doubtful one to the Austrian physician, as three cases, between five and ten years, proved it to be of but short duration, though the somewhat high mortality of 18.18 per cent. may be accounted for by the other extreme in the time-table, which many of the cases had attained. But the high age of these individuals, as well as the very scarcity of the cases, when in the community so large a number of persons previously attacked, or repeatedly vaccinated, exist, induces us to attribute to such immunity more efficacy than Dr. Keller is willing to grant.

